# Inositol polyphosphate–protein interactions: Implications for microbial pathogenicity

**DOI:** 10.1111/cmi.13325

**Published:** 2021-03-25

**Authors:** Sophie Lev, Bethany Bowring, Desmarini Desmarini, Julianne Teresa Djordjevic

**Affiliations:** ^1^ Centre for Infectious Diseases and Microbiology The Westmead Institute for Medical Research Sydney New South Wales Australia; ^2^ Sydney Medical School‐Westmead University of Sydney Sydney New South Wales Australia; ^3^ Marie Bashir Institute for Infectious Diseases and Biosecurity University of Sydney Sydney New South Wales Australia

**Keywords:** fungal pathogens, inositol polyphosphate kinases, inositol polyphosphates, inositol pyrophosphates, microbial pathogenesis, protein modification

## Abstract

Inositol polyphosphates (IPs) and inositol pyrophosphates (PP–IPs) regulate diverse cellular processes in eukaryotic cells. IPs and PP–IPs are highly negatively charged and exert their biological effects by interacting with specific protein targets. Studies performed predominantly in mammalian cells and model yeasts have shown that IPs and PP–IPs modulate target function through allosteric regulation, by promoting intra‐ and intermolecular stabilization and, in the case of PP–IPs, by donating a phosphate from their pyrophosphate (PP) group to the target protein. Technological advances in genetics have extended studies of IP function to microbial pathogens and demonstrated that disrupting PP–IP biosynthesis and PP–IP‐protein interaction has a profound impact on pathogenicity. This review summarises the complexity of IP‐mediated regulation in eukaryotes, including microbial pathogens. It also highlights examples of poor conservation of IP–protein interaction outcome despite the presence of conserved IP‐binding domains in eukaryotic proteomes.

## 1 INTRODUCTION

IPs and PP–IPs are produced by a series of sequentially acting IP kinases (IPKs). Using genetic and pharmacological approaches to modulate IP kinase (IPK) activity in conjunction with gel‐, HPLC‐ and, more recently, mass spectrometry–based metabolic profiling strategies, the identification and role of IPs and PP–IPs were initially elucidated in mammalian cells and the model yeasts, *Saccharomyces cerevisiae* and *Schizosaccharomyces pombe*. IPK enzymatic function has also been confirmed in vitro using chemically synthesized substrates. These studies revealed that IPs and PP–IPs function in a diverse range of cellular processes including glucose homeostasis, insulin sensitivity and secretion, fat metabolism and cellular energy dynamics, growth factor signalling, phosphate homeostasis, vesicular trafficking, DNA damage and repair, chromatin remodelling, spermatogenesis, neuronal migration, neutrophil activity, aging, apoptosis and platelet function (Lee, Kim, Ahn, & Kim, 2020). Hence, it is not surprising that dysregulated IP and PP–IP biosynthesis in human cells is associated with numerous diseases including Huntington's disease (Ahmed et al., 2015), diabetes and obesity (Chakraborty et al., 2010) and cancer (Rao et al., 2015).

Advances in genome sequencing and genome manipulation technology have subsequently allowed investigation into the roles of IPs and PP–IPs in microbial pathogenicity. The most significant progress has been in: *Cryptococcus neoformans*, an AIDS‐related fungal pathogen and the most common cause of fungal meningitis worldwide (Rajasingham et al., 2017); *Candida albicans*, the commensal and opportunistic nosocomial pathogen and most prevalent cause of fungal infections worldwide (Bongomin, Gago, Oladele, & Denning, 2017); *Trypanosoma brucei* and *T*. *cruzi*, the insect vector‐transmitted protozoan parasites causing sleeping sickness and Chagas disease, respectively; the human immunodeficiency virus (HIV) which attacks the immune system and leads to acquired immunodeficiency syndrome (AIDS) and *Clostridium difficile*, a multidrug‐resistant nosocomial bacterial pathogen that causes colitis. The importance of IPs and PP–PPs in these pathogens, as well as mechanistic insight into their mode of action at the molecular level, is discussed in more detail below.

## 2 EFFECTS OF DISRUPTING IP BIOSYNTHESIS ON MICROBIAL PATHOGENICITY

### 2.1 Cryptococcus neoformans

**FIGURE 1:**
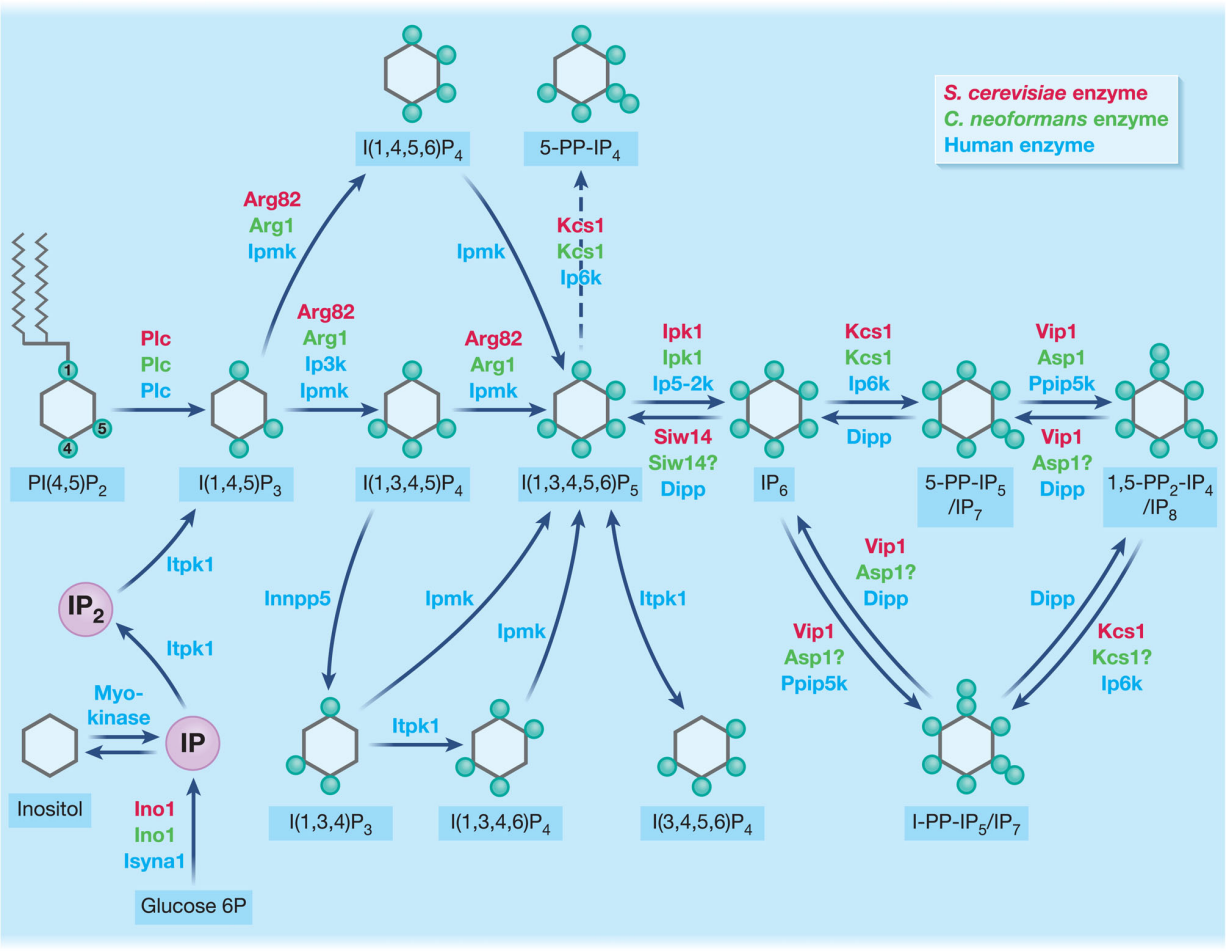
Diagram of the biosynthesis pathways of soluble IPs in humans, *S*. *cerevisiae* and *C*. *neoformans*, colour‐coded to differentiate each pathway. Dashed line: only occurs in absence of Ipk1 activity

Enzymes involved in IP and PP–IP biosynthesis were identified in *C*. *neoformans* by their homology to the corresponding enzymes in *S*. *cerevisiae* and deleted using homologous recombination (Lev et al., 2013; Lev et al., 2015; Li et al., 2017; Li et al., 2016a). A comparison of the metabolic profiles of the WT and deletion mutant strains revealed that synthesis of IP and PP–IPs is initiated by phospholipase C1 (Plc1)‐mediated hydrolysis of membranal phosphatidylinositol 4,5‐bisphosphate (PIP_2_), which generates I(1,4,5)P_3_. IP_3_ is further phosphorylated by the dual‐specificity IPK, Arg1, to I(1,4,5,6)P_4_ and I(1,3,4,5,6)P_5_. Ipk1 converts IP_5_ to fully phosphorylated I(1,2,3,4,5,6)P_6_. IP_6_ is the precursor of inositol pyrophosphates (PP–IPs), which have one or two covalently attached di(pyro)phosphates. Specifically, IP_6_ is further phosphorylated at position 5 by the hexakisphosphate kinase, Kcs1, to produce 5‐PP‐IP_5_ (IP_7_). Kcs1 is also an IP_5_ kinase producing PP‐IP_4_. However, the physiological relevance of PP‐IP_4_ in *C*. *neoformans* is unknown as this PP–IP is only observed in the absence of Ipk1 activity (Li et al., 2016a). IP_7_ is phosphorylated by Asp1 to form 1,5‐PP_2_‐IP_4_ (IP_8_) where the pyrophosphate groups are on position 1 and 5. The IP biosynthesis pathways in *C*. *neoformans* and *S*. *cerevisiae* are similar and less complex compared to the mammalian pathway (Figure 1). Two IP_3_ kinase homologues were identified in *C*. *neoformans*: Arg1 and Arg2, which share 22% and 15% identity, respectively, with Arg82 and 17% identity with each other (Lev et al., 2013). Both Arg1 and Arg2 contain a conserved PDKG motif essential for the catalytic activity of IP_3_ kinases. However, only Arg1 has IP_3_ kinase activity in vivo (Lev et al., 2013), and the physiological relevance of Arg2 remains to be elucidated. In contrast to yeast cells, conversion of IP_3_ to IP_5_ in mammalian cells occurs via several routes involving four different IPKs, with inositol polyphosphate multikinase (IPMK) involved in all pathways (Figure 1). The synthetic redundancy in the early steps of the pathway, coupled with a low IPK sequence homology, highlights early steps in the IPK pathway as attractive targets for anti‐fungal drug development, due to the minimised chance of off‐target effects on the human host (Lev et al., 2019; Li et al., 2016b).

Like the Vip1/Asp1 homologues in *S*. *cerevisiae*/*S*. *pombe*, Asp1 of *C*. *neoformans* has the features of a bi‐functional enzyme, with an N‐terminal ATP grasp domain responsible for the IPK activity and a C‐terminal histidine acid phosphatase domain (Lev et al., 2015). Vip1/Asp1 homologues in model yeast control the levels of 1‐PP‐IP_5_ and 1,5‐PP_2_‐IP_4_ (IP_8_) via their synthesis (IPK domain) and destruction (acid phosphatase domain) and inositol pyrophosphate levels can be skewed in either direction by mutating individual catalytic sites (Dollins et al., 2020). Both the bi‐functionality of Asp1 and its ability to synthesize the 1‐PP‐IP_5_ isoform remain to be demonstrated in *C*. *neoformans*. Breakdown of inositol pyrophosphate can also occur via the diphosphoryl inositol polyphosphate phosphatase (DIPP) class of pyrophosphatase (Safrany et al., 1999). However, DIPPs are more promiscuous and hydrolyse nucleotide dimers and polyphosphates (Lonetti et al., 2011). DIPP homologues have been identified in *C*. *neoformans* but remain to be characterized.

Disrupting IP biosynthesis in *C*. *neoformans* leads to dramatically altered transcriptional profiles, numerous cellular defects and loss of virulence in mouse infection models (Lev et al., 2013; Lev et al., 2015; Li et al., 2016a; Li et al., 2017). The absence of IP_7_ (isoform 5‐PP‐IP_5_) has a greater impact on cellular function than absence of IP_6_ or IP_8_ (Lev et al., 2015; Li et al., 2016a). Cryptococcal 5‐PP‐IP_5_ deficiency coincides with a 37°C growth defect, defective mitochondria, inability to utilise alternative carbon sources, compromised cell wall integrity, diminished melanisation and reduced mannoprotein exposure at the cell surface (Lev et al., 2015). The latter coincides with a failure to elicit a strong immune response in vivo and in vitro. The polysaccharide capsule, which is a major virulence factor and diagnostic marker of this pathogen, was also altered in 5‐PP‐IP_5_‐deficient *C*. *neoformans*, being larger and more mucoid (Lev et al., 2015). Furthermore, the phosphate (PHO) signalling pathway failed to become activated in 5‐PP‐IP_5_‐deficient *C*. *neoformans* when cellular phosphate levels declined as discussed below. Thus, 5‐PP‐IP_5_ is required for *C*. *neoformans* to respond to host stress, undergo metabolic adaptation to the host environment and acquire phosphate. Infection with 5‐PP‐IP_5_‐deficient *C*. *neoformans* is asymptomatic, and the pathogen cannot disseminate from the lungs to the brain (Lev et al., 2015).

Inositol is important for the development and pathogenicity of *C*. *neoformans* and is a precursor for the synthesis of the Plc1 substrate, PIP_2_ and hence the generation of IP_3_. Liao et al. (2018) showed that PP–IP biosynthesis fine‐tunes inositol acquisition to maintain inositol homeostasis in *C*. *neoformans*. In contrast to *S*. *cerevisiae*, they found that PP–IP biosynthesis is dispensable for de novo synthesis of inositol in *C*. *neoformans*, consistent with the role of PP–IPs in inositol metabolism in *C*. *neoformans*, being distinct from that of *S*. *cerevisiae*.

The combined loss of IP_4‐7_ in the *arg1*Δ mutant results in a more exacerbated 37°C growth defect, reduced capsule size, enhanced recognition by phagocytes, thickened cell walls and enlarged vacuoles (Li, Lev, et al., 2017). Interestingly, the invasion‐promoting enzyme, phospholipase B1 (PLB1), was excessively N‐linked glycosylated, and this coincided with a blockage in PLB1 secretion. In contrast to infection with the 5‐PP‐IP_5_‐deficient *kcs1*Δ mutant, infection with the IP_4‐7_‐deficient *arg1*Δ mutant was cleared in a mouse infection model (Li, Lev, et al., 2017).

### 2.2 Candida albicans

Recent studies on IPK knockout strains in the opportunistic fungal pathogen, *C*. *albicans*, have begun to elucidate the role of IPs and PP–IPs in cellular function (Li, Zhang, et al., 2017; Peng, Yu, Liu, Ma, & Li, 2020; Zhu et al., 2020). However, as no metabolic profiling was performed, the roles of specific IPs and PP–IPs are only putative. Homozygous deletion of the putative IP_5_ kinase‐encoding gene, *IPK1*, to create *ipk1ΔΔ*, resulted in dysfunctional mitochondria, which coincided with down‐regulation of genes involved in mitochondrial function, particularly those associated with oxidative phosphorylation (Zhu et al., 2020). The *ipk1ΔΔ* mutant also had a fitness defect when grown on standard laboratory media and was hypersensitive to anti‐fungal drugs, oxidising agents, cell wall perturbing agents and macrophage‐induced killing and was attenuated for virulence in a mouse dissemination model (Zhu et al., 2020). The results implicate the importance of IP_6‐8_ for cellular functions required for pathogenicity.

The same group also evaluated the role of Kcs1, Vip1 and Ipk2 in *C*. *albicans* by creating the corresponding deletion mutants (Li, Zhang, et al., 2017; Peng et al., 2020). They found that Vip1 plays a more important role than Kcs1 in regulating energy metabolism but without damaging mitochondria. Specifically, they found that growth of the homozygous deletion mutant *vip1*ΔΔ, but not *kcs1*ΔΔ, was reduced in glucose‐containing medium, and that this coincided with an up‐regulation in glycolysis and down‐regulation in mitochondrial function. The glycolysis‐skewed metabolism was compensated for by an accumulation of lipid droplets (Peng et al., 2020). Only the *vip1*ΔΔ mutant accumulated cell wall chitin and exhibited plasma membrane leakage which eventually led to death. Neither the *vip1*ΔΔ nor the *kcs1*ΔΔ mutants were tested for virulence in animal models. The results suggest that, in contrast to *C*. *neoformans* (Lev et al., 2015) and *S*. *cerevisiae* (Lee, Mulugu, York, & O'Shea, 2007), IP_8_ is more important than IP_7_ in energy metabolism in *C*. *albicans*.

A homozygous *IPK2* deletion mutant could not be created in *C*. *albicans*, presumably because complete loss of *IPK2* function is lethal (Li, Zhang, et al., 2017). Instead, a conditional knock‐down approach was used to limit IP_4‐8_ biosynthesis. Similar to *C*. *neoformans*, IP deficiency impacted numerous cellular functions and coincided with altered gene expression and secretion (Li, Zhang, et al., 2017). Interestingly, virulence traits such as the secretion of hydrolytic enzymes involved in nutrient acquisition, invasion of host tissues and hyphal development were enhanced (Li, Zhang, et al., 2017). Enhanced hyphal development coincided with an increase in the expression of hypha‐specific genes and transport of hypha‐specific factors. Changes in Ca^2+^ homeostasis were also observed in a IP_4_‐_8_‐deficient conditional knock down strain, which would have elevated IP_3_. This is consistent with the hypothesis that IP_3_ influences the activity of calcium channels in the vacuole as discussed below. Given that animal studies were unable to be conducted with the conditional knock down mutant, phenotypes observed in vitro were unable to be correlated with virulence in animal models. The results suggest that loss of IP_4‐8_ has a more dramatic impact on cellular function in *C*. *albicans* than loss of IP_6‐8_.

### 2.3 Trypanosoma species

The parasite *T*. *brucei* is transmitted between vertebrates, including humans, by the tsetse fly and causes African sleeping sickness. Similar to *C*. *albicans*, a conditional knock‐down approach was used in *T*. *brucei* to study IP function, and the results demonstrated that almost every IP conversion step is essential for parasite growth and infectivity (Cestari, Haas, Moretti, Schenkman, & Stuart, 2016). For example, IP_3_‐mediated calcium homeostasis is essential for growth and infectivity of *T*. *brucei*. (Huang, Bartlett, Thomas, Moreno, & Docampo, 2013).

*Trypanosoma cruzi*, another insect‐vector‐transmitted protozoan parasite, causes Chagas disease (American trypanosomiasis), and its IP biosynthesis pathway also regulates its differentiation and infectivity (Hashimoto et al., 2013; Mantilla, Amaral, Jessen, & Docampo, 2021). In a recent study, Mantilla, Amaral, et al. (2021) dissected the contribution of IPs and PP–IPs to the life cycle stages of *T*. *cruzi* (epimastigotes, cell‐derived trypomastigotes and amastigotes). Their combined use of reverse genetics and liquid chromatography mass spectrometry revealed the presence of IP_6_, IP_7_ and IP_8_. These species were not detected previously by HPLC analyses of cell lysates containing products of exogenously administered, radio‐labelled inositol, and suggest that IP_6‐8_ is derived from an endogenous source of inositol. The kinases involved in IP synthesis, TcIPMK, TcIP5K and TcIP6K, were also identified. In contrast to *T*. *brucei*, the TcIPMK knockout strain was viable; hence, the TcIPMK gene is dispensable in *T*. *cruzi* epimastigotes. However, TcIPMK was critical for virulence of the infective stages. The detection of highly phosphorylated IPs in TcIPMK knockout cells suggests that endogenous inositol is utilized for their synthesis. In contrast to *T*. *brucei*, TcIP5K was essential for survival of *T*. *cruzi* epimastigotes, consistent with the critical importance of IP_6‐8_. In another recent study, Mantilla, Amaral, et al. (2021) revealed 5‐IP_7_‐regulated processes in the two proliferative stages of *T*. *cruzi*, which is discussed in the IP mechanism section below on pyrophosphorylation.

IPs also contribute to *T*. *cruzi* pathogenicity through the biosynthesis of glycosylphosphatidylinositol (GPI) membrane anchors, which attach variant surface glycoproteins (VSG). The periodic switching of VSG in *Trypanosoma* species helps the parasite evade clearance by the host immune system (Cestari & Stuart, 2015). Another parasite genus of medical importance, *Leishmania*, also utilises GPI anchors to tether surface molecules to the plasma membrane (Forestier, Gao, & Boons, 2014). GPI anchors are synthesized from glucose, with glucose 6‐phosphate being converted to inositol‐3‐phosphate by the inositol‐3‐phosphate synthase, Ino1 (Figure 1). Inositol‐3‐phosphate is dephosphorylated by inositol monophosphatase, generating *myo*‐inositol. Inositol is utilised by phosphatidylinositol (PI) synthase to produce PI, which is preferentially used for the synthesis of GPI anchors (Martin & Smith, 2006).

### 2.4 Human Immunodeficiency Virus

HIV is a retrovirus with only a small genome and does not encode IP biosynthetic machinery. However, the HIV virus uses IPs synthesized by the host cell to promote viral replication. Recent studies have demonstrated a critical role for host‐derived IP_6_ as an inter‐molecular stabilising agent involved in both the maturation and replication of the HIV virion. The role of IPs as intermolecular glue is discussed in more detail below. It has also been shown that IP_5_ can substitute for IP_6_ as the intermolecular stabilising agent when conversion of IP_5_ to IP_6_ is blocked in infected cells. Specifically, host‐derived IP_6_ plays a role in capsid assembly. IP_6_ binding increases HIV‐1 capsid stability from minutes to hours and promotes DNA accumulation inside intact structures during reverse transcription (Mallery et al., 2018; Marquez et al., 2018). Conversely, HIV within IP_6_‐deficient cells produces unstable capsids and fewer virions, while virions that fail to bind sufficient IP_6_ are poorly infectious and fail to replicate in primary cells (Mallery et al., 2019).

### 2.5 Clostridium difficile

*Clostridium difficile* is a common, life‐threatening nosocomial pathogen, which has become resistant to multiple antibiotics (Ananthakrishnan, 2011; He et al., 2013). Infection symptoms and virulence are mediated by the protein Toxin B (TcdB), which is secreted into the gut lumen (Lyras et al., 2009). TcdB becomes a virulence factor following its receptor‐mediated endocytosis into cells lining the host gut lumen. Post endocytosis, the cysteine–protease domain in TcdB undergoes a pH‐dependent conformational change, exposing a site for binding of the host‐derived cofactor, IP_6_, which allosterically regulates zinc‐dependent TcdB auto‐proteolysis, releasing the harmful enzymatic domain (Chumbler et al., 2016; Egerer, Giesemann, Herrmann, & Aktories, 2009; Pruitt et al., 2009; Reineke et al., 2007). IP_6_ stabilizes the active conformation by binding the positively charged binding pocket on the cysteine–protease domain (Chumbler et al., 2016; Shen et al., 2011). Ivarsson et al. (2019) have shown that IP_6_ triggers auto‐processing of TcdB in vitro, but not in the presence of luminal concentrations of calcium (>10 mM). Toxin cleavage becomes blocked because IP_6_ forms insoluble complexes with calcium (Shears, 2001). To overcome this, Ivarsson et al. (2019) synthesized IP_6_ analogues where some phosphate moieties are replaced with sulfate and demonstrated that they retain their allosteric activity in vitro but are less susceptible to forming insoluble complexes with Ca^2+^. IP_6_ analogues are currently being pursued as a therapeutic approach, aimed at triggering pre‐emptive auto‐proteolysis of TcdB in the gut lumen, preventing internalization of the harmful enzymatic domain. Altering the location of toxin cleavage from inside cells to the intestinal lumen circumvents the need for drug absorption and provides a potentially superior approach to classical inhibition of the protease domain. Oral administration of IP_6_ analogues reduced inflammation and promoted survival in a mouse model of *C*. *difficile* infection (Ivarsson et al., 2019).

## 3 MECHANISTIC INSIGHT INTO THE BIOLOGICAL ROLES OF IPS AT THE MOLECULAR LEVEL

**FIGURE 2:**
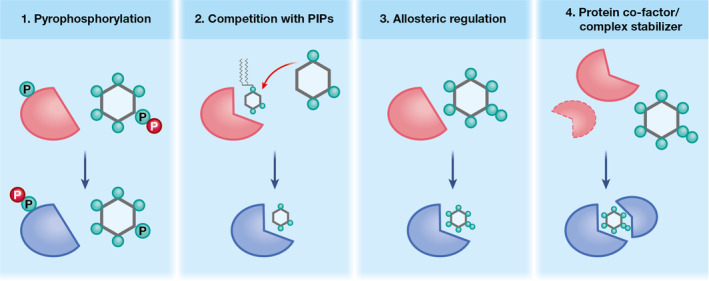
Summary of the different modes of IP/PP‐IP regulation of the target protein. **1**. Pyrophosphorylation which involves donation of a phosphate from the pyrophosphate group (PP) of a PP‐IP to a prephosphorylated serine/threonine on the target protein; **2**. Competition with lipid‐based phosphatidylinositols (PIPs) which displaces the target protein off the membrane; **3**. Allosteric regulation which is associated with a conformational change in the target protein and **4**. Protein co‐factor or complex stabilizer to promote intra‐ and intermolecular stabilization of target protein(s)

Due to their high phosphate density, IPs and PP–IPs are highly negatively charged under physiological conditions and can interact with positively charged regions in their target proteins. IPs and PP–IPs have four major biological roles: allosteric regulation where the binding of the IP/PP–IP induces a conformational change in the target protein to modulate its activity; competition with lipid‐based inositol phosphates (phosphoinositides) to modulate membrane‐associated signalling processes; structural cofactor to stabilise proteins and protein–protein interactions and protein pyrophosphorylation which can only be performed by inositol pyrophosphates. The broad range of IP species and target proteins, as well as fluctuations in IP abundance and subcellular compartmentalization, contribute to the diversity of IP‐regulated functions. To further add to the complexity of IP‐mediated regulation in eukaryotes, the physiological effects of IP binding to their target proteins often differ, even at the species level. A summary of the biological roles of IPs is shown in Figure 2.

### 3.1 Pyrophosphorylation‐dependent effects of PP–IPs on protein target function

Inositol pyrophosphates (PP–IPs) are high‐energy phosphate metabolites that can donate the terminal phosphate of their pyrophosphate moiety to a pre‐phosphorylated serine or threonine on a target protein in a β‐phosphoryl transfer reaction (Bhandari et al., 2007). Pyrophosphorylation is an unusual modification in that it is enzyme‐ and ATP‐independent. It also requires Mg^2+^ as a cofactor and ‘priming’ by a protein kinase, commonly casein kinase 2 CK2. 5‐PP‐IP_5_ affinity chromatography and mass spectrometry have been used to identify the IP_7_ interactome in *S*. *cerevisiae* and more recently in *T*. *cruzi* (Mantilla, Kalesh, Brown Jr, Fiedler, & Docampo, 2021; Wu, Chong, Perlman, Resnick, & Fiedler, 2016) using a pull‐down buffer with or without magnesium to distinguish proteins that bind IP_7_ from those that become pyrophosphorylated by IP_7_, respectively. In the *T*. *cruzi* study, recently developed electron‐tandem mass spectrometry technology, involving transfer dissociation combined with higher energy collision dissociation (CID‐EThcD), enabled reliable and unambiguous assignment of endogenous peptides containing serine and threonine pyrophosphorylation, distinguishing them from peptides containing two individual phosphorylated amino acids (Penkert et al., 2017). Using this technology, a choline/*o*‐acetyltransferase domain‐containing phosphoprotein that undergoes 5‐IP_7_‐mediated phosphorylation events at a polyserine tract (Ser578‐580) was identified.

IP_7_ regulates dynein‐driven vesicular movement by pyrophosphorylating the N‐terminus of the dynein intermediate chain to promote dynein–dynactin interaction in mammalian cells (Chanduri et al., 2016). Pyrophosphorylation is also prevalent in the nucleolus. In yeast, pyrophosphorylated proteins include the ribosomal chaperone, SRP40 and NSR1, which is involved in ribosome assembly and export (Bhandari et al., 2007; Saiardi, Bhandari, Resnick, Snowman, & Snyder, 2004). Despite the growing number of pyrophosphorylated proteins being discovered, the effect of pyrophosphorylation on many of these target proteins remains to be elucidated.

### 3.2 IPs regulate chromatin remodelling

Class I histone deacetylates (HDACs) are involved in chromatin remodelling. HDACs inactivate transcription by catalysing removal of acetyl groups from lysine residues on histones. I(1,4,5,6)P_4_ is present in the mammalian HDAC3:SMRT crystal structure, and I(1,4,5,6)P_4_ interaction enhances HDAC‐mediated transcriptional inactivation (Millard et al., 2013; Watson et al., 2016; Watson, Fairall, Santos, & Schwabe, 2012). Transcriptional inactivation occurs when HDAC is recruited to a SMRT/NCoR2 repression complex and interacts with the SANT (Swi3, Ada2, N‐Cor and TFIIIB) domain of SMRT. I(1,4,5,6)P_4_ enhances activity of the newly formed complex by making extensive contact with both proteins and becomes sandwiched within a highly basic binding pocket formed by both proteins. It was also reported that I(1,3,4,5,6)P_5_ and IP_6_ activate HDAC complexes to the same extent as I(1,4,5,6)P_4_ (Watson et al., 2016). Thus, IP binding to HDAC complexes facilitates both interaction of HDAC components and allosteric regulation of the complex.

The activity of mammalian HDAC1/2 in the context of another repression complex, Sin3L/Rpd3L, is also enhanced by IPs (IP_4_/IP_5_/IP_6_) in vitro, via IP interaction with the zinc finger domain of the SAP30L subunit, rather than the SANT domain (Marcum & Radhakrishnan, 2019). A SAP30L zinc finger domain (pfam 13,866) has not been identified in fungal proteomes, suggesting that zinc finger domain–IP interaction is unique to higher eukaryotes. While IP_4_, IP_5_ and IP_6_ enhance HDAC‐mediated transcriptional inactivation in mammalian cells, the inositol pyrophosphates 1‐PP‐IP_5_, 5‐PP‐IP_5_ and 5‐PP‐IP_4_ have been implicated in this role in the *S*. *cerevisiae* class I HDAC homologue, Rpd3L (Worley, Luo, & Capaldi, 2013). Key IP binding residues in human class I HDACs are almost entirely conserved in Rpd3 from *S*. *cerevisiae* suggesting that IP involvement in assembly and activation of class I HDACs is evolutionarily conserved in eukaryotes (Millard et al., 2013; Watson et al., 2012).

Another example of IP involvement in chromatin remodelling is the nucleosome remodelling complex, SWI/SNF, in *S*. *cerevisiae*. In this case, I(1,4,5,6)P_4_ and IP_5_ can stimulate nucleosome mobilisation by SWI/SNF (Shen, Xiao, Ranallo, Wu, & Wu, 2003). SWI/SNF uses ATP hydrolysis to slide nucleosomes along the DNA and expose it to transcription factors, contributing to transcriptional repression as well as activation (Roberts & Orkin, 2004). It is not known which of the nine proteins in the SWI/SNF complex are involved in IP binding.

### 3.3 IP competition with phosphoinositides for binding to PH domains

Despite their low abundance, phosphoinositides play key roles in regulating cellular function by tethering specific types of cellular proteins to the membrane to control their compartmentalisation and often their activity. One example is the pleckstrin homology (PH) domain‐containing proteins, which are recruited to and regulated by phosphoinositides, including PIP_2_ and PIP_3_ (Hammond & Balla, 2015). Phosphoinositide–PH domain protein interactions regulate diverse functions including signalling, cytoskeletal organisation, vesicular trafficking, phospholipid processing and glucose homeostasis (Lenoir, Kufareva, Abagyan, & Overduin, 2015). Studies performed predominantly in mammalian cells have shown that, unlike other phosphoinositide‐binding domains, PH domains also bind soluble IPs, which are structurally similar to phosphoinositide headgroups and often with a similar affinity to phosphoinositides. Hence there is a growing body of evidence suggesting that IPs act as competitive regulators of PH domain–phosphoinositide interactions involved in cell signalling. In support of this, competition of IP_7_ with PIP_3_ for binding to the PH domain of Akt, a signalling kinase involved in glucose homeostasis, has been visualised in living cells following photochemical release of caged IP_7_ into the cytoplasm (Pavlovic et al., 2016). This competition would prevent Akt phosphorylation by membrane kinases.

IP‐based analogues are currently being pursued as inhibitors, particularly in the case of phosphoinositide 3‐kinase (PI3K)‐mediated signalling pathways, which have a well‐established role in cancer development and progression (Maffucci & Falasca, 2020). Similar to mammalian cells, yeast PH domain proteins bind PI(3)P, PI(4,5)P_2_, PI(3,4)P_2_ and PI(3,4,5)P_3_ to trigger physiological responses including actin regulation, membrane dynamics (Fadri, Daquinag, Wang, Xue, & Kunz, 2005) and spore development (Nakamura‐Kubo, Hirata, Shimoda, & Nakamura, 2011). Despite ample evidence for the importance of PIP_2_–PH domain protein interaction in yeast, potential competitive roles of IPs and PP–IPs in fine‐tuning these interactions are yet to be identified.

IP_3_ competition with phosphoinositides for binding to the PH domains of membrane proteins could be the reason why the IP_3_ kinase‐deficient (*arg1*Δ) mutant of *C*. *neoformans* has a more defective phenotype in vitro and in vivo than the IP_7_‐deficient (*ksc1*Δ) mutant of *C*. *neoformans*. Unlike IP_7_‐deficient *C*. *neoformans*, the *arg1*Δ mutant (deficient in IP_4‐7_) is unable to phosphorylate IP_3_ and, therefore, accumulates IP_3_. This excess IP_3_ could potentially alter membrane and signalling functions by competing with membrane‐localised phosphoinositides for binding to their PH domain proteins (Lemmon & Ferguson, 2000). Alternatively, excess IP_3_ could inhibit the activity of its membrane‐associated progenitor, Plc1, via a feedback inhibition loop, since the major substrate of Plc1 in *C*. *neoformans* is the phosphoinositide, PIP_2_ (Lev et al., 2013). Support for the latter is that both the *arg1*Δ and *plc1*Δ mutants share all the phenotypes that are absent in IP_7_‐deficient strains and accumulate PIP_2_ (Lev et al., 2013).

### 3.4 IP competition in mRNA decapping

The 5‐PP‐IP_5_ isomer of IP_7_ has recently been demonstrated to regulate mRNA stability and the dynamics of P‐bodies, which are purportedly the sites for sequestration and storage of mRNAs away from the translating pool and where mRNA decay occurs (Sahu et al., 2020). By modulating 5‐PP‐IP_5_ levels genetically and pharmacologically, this study demonstrated that 5‐PP‐IP_5_ competes with 5′‐capped mRNA for hydrolysis by NUDT3, a DIPP1 which dephosphorylates all PP‐IPs, including 5‐PP‐IP_5_, and thereby impacts cellular mRNA transcript levels. The study also reported that P‐body abundance changed in accordance with the 5‐PP‐IP_5_–modulated levels of NUDT3‐regulated mRNA transcripts.

### 3.5 IP roles in calcium homeostasis

A *C*. *albicans* conditional mutant, predicted to have reduced levels of IP_4‐8_ and an excess of IP_3_, has an elevated level of Ca^2+^ (Li, Zhang, et al., 2017). It is known that IP_3_ spikes in higher eukaryotes in response to external stimuli and that IP_3_ binds to IP_3_‐gated calcium channels in the endoplasmic reticulum (ER) to trigger a transient influx of Ca^2+^ into the cytosol (Foskett, White, Cheung, & Mak, 2007). However, the source of the elevated intracellular Ca^2+^ in *C*. *albicans* is not known. Several studies demonstrated an IP_3_‐dependent increase in cytosolic calcium in *S*. *cerevisiae*. However, IP_3_‐gated ER calcium channel orthologues have not been identified in fungal genomes (Alzayady et al., 2015; Tisi et al., 2004) suggesting that the source of the intracellular Ca^2+^ is not the ER. In yeast, the vacuole, rather than the ER, is the most important calcium storage compartment, with Ca^2+^ homeostasis regulated by a Ca^2 +^ ‐ATPase (Pmc1), a Ca^2+^/H^+^ exchanger (antiport) (Vcx1) and a calcium channel homologue of the transient receptor potential channels (Yvc1) (Palmer et al., 2001). Yvc1 mediates Ca^2+^ efflux from the vacuole to the cytoplasm under conditions of stress (Denis & Cyert, 2002; Zhou et al., 2003). Evidence obtained in *S*. *cerevisiae* using an *IPK2/YVC1* double mutant suggests that IP_3_ could interact directly, or indirectly, with Yvc1 to control its opening and trigger Ca^2+^ signalling in the cytosol (Bouillet et al., 2012). Whether IP_3_ has the same function in fungal pathogens remains to be elucidated.

### 3.6 IP roles as intermolecular stabilisers in HIV pathogenicity

In addition to regulating protein activity, IPs function as intermolecular stabilisers within multiprotein complexes. One example is during HIV pathogenesis where the replicating virus utilises IP_6_ from the host cell to stabilise its capsid and promote the assembly and maturation of infectious virions (Dick, Mallery, Vogt, & James, 2018; Mallery et al., 2018; Mallery et al., 2019; Marquez et al., 2018). IP_6_ specifically interacts with two lysine residues (K158 and K227) in the immature Gag hexamer and assists in driving the formation of the immature lattice in HIV. This once again demonstrates the importance of electrostatic interactions between IPs and positively charged residues in target proteins. The highly conserved immature lattice lysine rings, K158 and K227, and mature capsid charged ring (e.g., R18) across diverse lentiviruses suggest that IP_6_ is essential for lentiviral replication in general. Furthermore, Azevedo, Burton, Ruiz‐Mateos, Marsh, and Saiardi (2009) showed that IP_7_ (5‐PP‐IP_5_)‐mediated pyrophosphorylation of AP3B1, a clathrin‐associated protein complex required for HIV‐1 Gag release from HeLa cells, modulates AP3B1 interaction with a motor protein of the kinesin superfamily, Kif3A, which is also required for HIV‐1 Gag release, and consequently affects release of HIV‐1 virus‐like particles.

### 3.7 IP roles as allosteric regulators and intermolecular stabilisers in transcriptional and cell cycle regulation

Crystal structure analysis revealed that IP_6_ binds to the catalytic domain of human ADAR2, an RNA editing enzyme (Macbeth et al., 2005). IP_6_ is buried within the enzyme core and contributes to the protein fold and is required for enzyme activity. IP_6_ was also found to be essential for deamination of adenosine 37 of tRNA^ala^ by ADAT1 in vivo and in vitro (Macbeth et al., 2005).

IP_6_ promotes the formation of Cullin‐RING ligase (CRL)‐COP9 signalosome (CSN) complexes by acting as a CSN cofactor/intermolecular glue, recruiting CRL. CRLs are the largest family of ubiquitin E3s activated by neddylation and regulated by the deneddylase, CSN, and they mediate ubiquitylation of numerous proteins (Lin et al., 2020; Scherer et al., 2016). Crystal structure analysis revealed that IP_6_ binding to a cognate pocket formed by conserved lysine residues, strengthens CRL‐CSN interactions to dislodge the E2 CDC34/UBE2R from CRL and promote CRL deneddylation, thereby regulating signalosome interactions and CRL function. The key lysine residues in the IP_6_‐binding pocket, which are contributed by different components in the complex, are conserved in yeast, plants and humans. IP_6_ is therefore an essential conserved signalosome factor in evolutionarily distant organisms.

### 3.8 IP roles as an allosteric regulator and intermolecular stabiliser in phosphate homeostasis via SPX domain interaction

Syg1‐Pho81‐Xrp1 (SPX) domains occur in mammalian, plant and fungal proteins and have roles in phosphate homeostasis via their ability to bind PP–IPs (Wild et al., 2016). Using site‐directed mutagenesis and X‐ray crystallography, Wild et al. (2016) identified a positively charged binding pocket on the surface of SPX domains from mammalian, plant and fungal proteins and showed that it binds IP_6_, IP_7_ and IP_8_ with high affinity and phosphate with low affinity. Their studies revealed that IP binding triggers a conformational change within the SPX domain to provide allosteric regulation. Based on a number of observations, Wild et al. (2016) concluded that PP–IPs are the physiologically relevant signalling molecules in yeast, humans and plants, signalling cellular phosphate status by binding to SPX domains in phosphate‐sufficient conditions and enabling them to interact with an array of proteins that regulate phosphate homeostasis.

Recent headway has been made into determining the hierarchy of importance of PP–IPs in phosphate homeostasis: 1‐PP‐IP_5_ mediates adaptations to phosphate starvation in *S*. *cerevisiae* (Lee et al., 2007); 5‐PP‐IP_5_ is the dominant activator of polyphosphate synthesis by the vacuolar transporter chaperone in *S*. *cerevisiae* (Gerasimaite et al., 2017; Wild et al., 2016), and 5‐PP‐IP_5_ stimulates Na^+^/phosphate cotransport by Pho91 in *T*. *brucei* (Potapenko et al., 2018). Both 5‐PP‐IP_5_ and IP_8_ have been implicated in regulating XPR1‐driven phosphate efflux in human cells (Li et al., 2020; Wilson, Jessen, & Saiardi, 2019). However, using a number of different strategies including liposome‐mediated delivery of metabolically resistant phosphate‐carbon‐phosphate (PCP) analogues of PP–IPs into cells, (Li et al., 2020) found that the hierarchy of importance favours IP_8_ over 5‐PP‐IP_5_ and 1‐PP‐IP_5_ in human cells. This finding is supported by recent work in the model plant, *Arabidopsis thaliana*, where IP_8_ was found to be essential for initiating a phosphate starvation response by promoting intermolecular interaction between the stand‐alone SPX domain protein, SPX1, considered to be a phosphate sensor, and the central regulator of the phosphate starvation responses, PHR1 (Dong et al., 2019; Zhu et al., 2019). IP_8_ deficiency had no reported effects on phosphate homeostasis in *S*. *cerevisiae* or *C*. *neoformans* (Desmarini et al., 2020).

A recent study has shed light on the regulatory role of PP–IPs in phosphate homeostasis in the fungal pathogen *C*. *neoformans*. *C*. *neoformans* activates its phosphate (PHO) signalling pathway via the transcription factor Pho4, which is essential for its pathogenicity (Lev et al., 2017). This is consistent with the pathogen experiencing phosphate deprivation during infection. In *S*. *cerevisiae* and *C*. *neoformans*, PHO pathway activation depends on Pho81, a cyclin‐dependent protein kinase (CDK) inhibitor containing an SPX domain (Desmarini et al., 2020; Toh‐e et al., 2015). Pho81 forms a trimeric complex with the CDK, Pho85, and its associated cyclin, Pho80, which directs Pho85 to phosphorylate its substrate, Pho4. Pho81‐Pho80‐Pho85 forms a complex in *S*. *cerevisiae* and *C*. *neoformans* irrespective of phosphate status (Desmarini et al., 2020; Schneider, Smith, & O'Shea, 1994). During phosphate deprivation, Pho81 inhibits Pho85 and prevents it from phosphorylating Pho4. Unphosphorylated Pho4 is retained in the nucleus where it promotes transcription of genes involved in phosphate acquisition (Lev et al., 2017). In *S*. *cerevisiae* the IP_7_ isoform, 1‐PP‐IP_5_, allosterically regulates the SPX domain of Pho81 to trigger PHO pathway activation (Lee et al., 2007). In contrast, the 5‐PP‐IP_5_ isoform regulates PHO pathway activation in *C*. *neoformans* (Desmarini et al., 2020). In this study, a conserved lysine surface cluster, K^221,224,228^, was identified in the SPX domain of Pho81 and demonstrated to be important for binding 5‐PP‐IP_5_ (Desmarini et al., 2020). Similar to *PHO4* deletion, disrupting 5‐PP‐IP_5_ interaction with the SPX domain of Pho81 prevented PHO pathway activation in phosphate‐starved *C*. *neoformans* but led to avirulence, rather than attenuated virulence, in a mouse infection model (Desmarini et al., 2020). The reduction in virulence is consistent with the IP_7_‐regulated CDK complex having functions that extend beyond the regulation of phosphate homeostasis.

It was proposed that 1‐PP‐IP_5_ has a role in phosphate sensing in *S*. *cerevisiae*, as its levels increase during phosphate deprivation (Lee et al., 2007). In contrast, the levels of 5‐PP‐IP_5_ decrease in *C*. *neoformans* during phosphate deprivation (Desmarini et al., 2020). Despite this reduction, the remaining 5‐PP‐IP_5_ functions as intermolecular ‘glue’ to stabilise the association of Pho81 with Pho85 and its cyclin Pho80. Furthermore, 5‐PP‐IP_5_ binding‐defective Pho81 and native Pho81 are degraded during phosphate starvation and IP_7_ deficiency, respectively (Desmarini et al., 2020). This suggests that Pho81 stability is dependent upon its association with Pho80‐Pho85. Further evidence of the differing roles of IP_7_ isoforms in *S*. *cerevisiae* and *C*. *neoformans* is that the PHO pathway is hyperactivated in 5‐PP‐IP_5_‐deficient *S*. *cerevisiae* irrespective of phosphate availability (Auesukaree, Tochio, Shirakawa, Kaneko, & Harashima, 2005; Desmarini et al., 2020), while 5‐PP‐IP_5_ deficiency prevents PHO pathway activation in *C*. *neoformans* following phosphate deprivation (Desmarini et al., 2020). Thus, in contrast to human cells, plants and *S*. *cerevisiae*, the hierarchy of PP–IP importance in phosphate homeostasis favours 5‐PP‐IP_5_ in *C*. *neoformans*. Taken together, the literature reveals that even in relatively closely related species, IP_7_‐target protein duos are not conserved and have evolved to produce a different regulatory outcome for the same function.

It is tempting to speculate that the multiple phosphorylation sites displayed by IP_7_ to components of a CDK complex provide a biological alternative to multisite phosphorylation by various kinases. The latter has been proposed for the interaction of the CDK inhibitor, Cip1 (the putative homologue of mammalian p21), with Cdk1 and the cyclin Cln2 to promote cell cycle progression through G1 in *S*. *cerevisiae* (Chang et al., 2017). Cip1 becomes phosphorylated at three positions by the kinase Hog1 under hyperosmotic stress, and this phosphorylation is hypothesised to strengthen Cip1 and Cdk1–G1 cyclin interaction and induce transitory cell cycle arrest. Although IP_7_ has a proven role in stabilising the phosphate responsive CDK complex in *C*. *neoformans*, it cannot be ruled out that additional kinase‐induced phosphorylation of Pho81 contributes to CDK complex stabilisation. For example, in *S*. *cerevisiae*, Pho81 is a substrate of its own binding partner, Pho85.

## 4 CONCLUSIONS

Similar to mammalian cells and model yeast, dysregulated IP biosynthesis in pathogenic fungi and parasites leads to numerous cellular defects and major changes in the transcriptional profile. Studies in mammalian cells and yeast have provided much needed mechanistic insight into the biological roles of IPs and the outcomes of IP–protein interactions. A major theme that has emerged is that IPs and PP–IPs are key components of multisubunit complexes where they function as intermolecular glue to stabilise the complex and/or modulate complex activity. Via these associations, they are involved in the regulation of a diverse range of cellular functions. These studies have paved the way for elucidation of the roles IPs play as intermolecular stabilisers in microbial pathogens and promotion of an understanding that, despite the presence of conserved IP‐binding domains in eukaryotic proteomes, IP‐target protein duos are not conserved and have evolved to produce a different regulatory outcome, even in closely related species. Recent advances in methods used to concentrate and detect IP target proteins using stable, synthetic and conjugated IP analogues and to detect pyrophosphorylation targets using sophisticated mass spectrometry, will expedite the discovery of more novel IP targets and IP–target interaction in cellular function.

## CONFLICT OF INTEREST

The authors declare no conflict of interest.

## Data Availability

Data sharing is not applicable to this article as no new data were created or analysed in this study.
